# Boosting Tissue Vascularization: Nanofat as a Potential Source of Functional Microvessel Segments

**DOI:** 10.3389/fbioe.2022.820835

**Published:** 2022-02-04

**Authors:** Andrea Weinzierl, Yves Harder, Daniel Schmauss, Michael D. Menger, Matthias W. Laschke

**Affiliations:** ^1^ Institute for Clinical & Experimental Surgery, Saarland University, Homburg, Germany; ^2^ Department of Plastic, Reconstructive and Aesthetic Surgery, Ospedale Regionale di Lugano, Ente Ospedaliero Cantonale (EOC), Lugano, Switzerland; ^3^ Faculty of Biomedical Sciences, Università Della Svizzera Italiana, Lugano, Switzerland

**Keywords:** fat graft, nanofat, vascularization, dorsal skinfold chamber, intravital fluorescence microscopy, tissue engineering

## Abstract

Nanofat is increasingly applied in plastic surgery for the improvement of scar quality and skin rejuvenation. However, little is known about the underlying regenerative mechanisms. Therefore, we herein investigated nanofat grafts in a murine dorsal skinfold chamber model. Nanofat generated from subcutaneous, inguinal adipose tissue of green fluorescent protein (GFP)^+^ C57BL/6 male and female donor mice was injected intracutaneously into dorsal skinfold chambers of gender-matched GFP^−^ wild-type mice. The vascularization and tissue composition of the grafted nanofat were analyzed by means of intravital fluorescence microscopy, histology and immunohistochemistry over an observation period of 14 days. The freshly generated nanofat consisted of small fragments of perilipin^+^ adipocytes surrounded by Sirius red^+^ collagen fibers and still contained intact CD31^+^/GFP^+^ vessel segments. After transplantation into the dorsal skinfold chamber, these vessel segments survived and developed interconnections to the surrounding CD31^+^/GFP^−^ host microvasculature. Accordingly, the grafted nanofat rapidly vascularized and formed new microvascular networks with a high functional microvessel density on day 14 without marked differences between male and female mice. Even though further research is needed to confirm these findings, the present study suggests that nanofat boosts tissue vascularization. Thus, nanofat may represent a versatile resource for many applications in tissue engineering and regenerative medicine.

## Introduction

The first recorded fat graft was executed in 1893 by the German surgeon Gustav Neuber to correct adherent osteomyelitis-induced scar sequelae (Mazzola and Mazzola, 2015). After decades of using mainly *en bloc* fat grafts for the correction of contour deformities, the introduction of liposuction (Illouz, 1986) in the 1980s finally paved the way for modern fat grafting by means of easily reinjectable, aspirated microfat. Since then, microfat grafts have also been increasingly used due to their beneficial effects on damaged tissue, such as burn scars (Mazzola and Mazzola, 2015). The true regenerative potential of fat grafts was ultimately confirmed in the early 2000s, when the presence of mesenchymal stem cells was discovered in adult adipose tissue (Zuk et al., 2001; Zuk et al., 2002). Nowadays, microfat grafts are frequently used for breast reconstruction. However, this procedure bears the risk of graft absorption due to an insufficient vascularization.

In 2013, Tonnard et al. (2013) introduced the technique of nanofat grafting. This processing method uses emulsification and filtering of microfat to burst the majority of mature adipocytes, resulting in grafts that mostly consist of released growth factors and adipose-derived stem cells (ASCs). Hence, the principal aim of nanofat grafting is not the restoration of volume, but the induction of tissue remodeling and the improvement of tissue quality. In this context, it should be considered that adipose tissue does not only contain adipocytes and ASCs, but also various other cell types, such as fibroblasts and immune cells, as well as extracellular matrix and microvessels (Brüggen and Stingl, 2020; Chait and den Hartigh, 2020; Herold and Kalucka, 2021). However, the effects of these other components on the engraftment of nanofat remain largely unknown. Since vascularization is a major determinant for adequate tissue regeneration (Banfi et al., 2018; Laschke and Vollmar, 2018; Gianni-Barrera et al., 2020), it may be particularly interesting to study the fate of microvessels during the process of nanofat generation and transplantation. Given that the fabrication of nanofat only applies mechanical forces to break down the tissue, it is unlikely that individual cell-cell bonds are broken, as it is the case for the enzymatic digestion of adipose tissue into single stromal vascular fraction (SVF) cells (Zuk et al., 2001). Therefore, we herein hypothesized that nanofat still contains functional vessel segments that are able to rapidly reassemble into new microvascular networks.

It is well known that estrogen is a major promotor for angiogenesis and tissue vascularization (Rubanyi et al., 2002). In the context of fat grafting, this is demonstrated by the fact that fat grafts from non-ovariectomized mice are more vascularized when compared to ovariectomized animals (Bills et al., 2015). Furthermore, Mok et al. (2018) reported that elevated serum estrogen increases ASC density and early vascularization in microfat grafts. Similar results were observed by Wu et al. (2020), who detected a significantly higher expression of estrogen receptor α and a concomitantly elevated vascular density in grafted adipose tissue from animals receiving estradiol treatment. Based on these findings, we further speculated that the vascularization of grafted nanofat is improved in females.

To test our hypotheses in the present study, we generated nanofat from subcutaneous, inguinal adipose tissue of green fluorescent protein (GFP)^+^ C57BL/6 male and female donor mice. The nanofat was subsequently injected intracutaneously into dorsal skinfold chambers of gender-matched GFP^−^ wildtype animals to assess its vascularization and tissue composition by means of intravital fluorescence microscopy, histology and immunohistochemistry over an observation period of 14 days.

## Materials and Methods

### Animals

All animal experiments were approved by the local governmental animal protection committee (permit number: 30/2020) and conducted in accordance with the European legislation on the protection of animals (Directive 2010/63/EU) and the NIH Guidelines on the Care and Use of Laboratory Animals (NIH publication #85–23 Rev. 1985).

In this study, 4 male and 4 female GFP^+^ mice (C57BL/6-Tg (CAG-EGFP)1Osb/J; The Jackson Laboratory, Bar Harbor, ME, United States) with an age of up to 52 weeks and a body weight of 28–32 g served as gender-matched fat donors for the generation of nanofat in sufficient amounts. The nanofat was grafted into the dorsal skinfold chambers of 8 male and 8 female GFP^−^ C57BL/6 wild-type mice (Institute for Clinical & Experimental Surgery, Saarland University, Homburg/Saar, Germany) with an age of 12–20 weeks and a body weight of 23–26 g. The GFP^+^/GFP^−^ cross-over design of the present study allowed the differentiation of GFP^+^ cells originating from the nanofat grafts and GFP^−^ cells from the host tissue within the dorsal skinfold chamber.

The animals were kept at a room temperature of 22–24°C and a 12-h day-night cycle. They had free access to standard pellet chow (Altromin, Lage, Germany) and tap water. Mice with a dorsal skinfold chamber were kept one per cage for the duration of the experiments.

### Anesthesia

Harvesting of adipose tissue, dorsal skinfold chamber implantation and intravital fluorescence microscopy were performed in general anesthesia induced by intraperitoneal injection of ketamine (100 mg/kg body weight; Ursotamin^®^; Serumwerke Bernburg, Bernburg, Germany) and xylazine (12 mg/kg body weight; Rompun^®^; Bayer, Leverkusen, Germany). All animals received a subcutaneous injection of 5 mg/kg carprofen (Rimadyl^®^; Zoetis Deutschland GmbH, Berlin, Germany) during the chamber implantation to prevent postoperative pain.

### Generation of Nanofat

**FIGURE 1:**
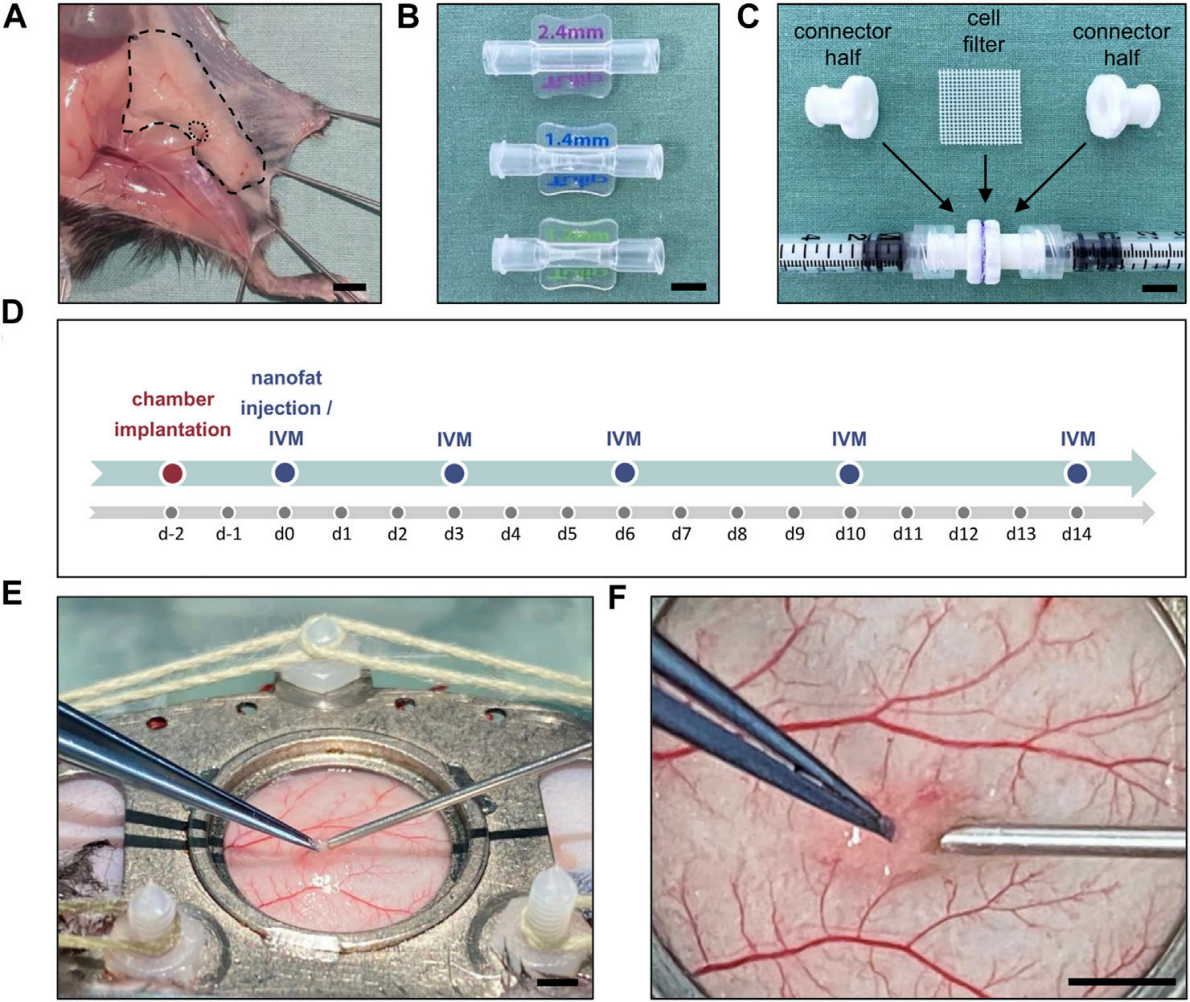
**(A)** Inguinal subcutaneous adipose tissue (borders marked by broken line) with its inguinal lymph node (borders marked by dotted line) for the generation of nanofat. Scale bar: 5 mm. **(B)** Luer lock connectors with descending internal diameters of 2.4 mm (top), 1.4 mm (middle) and 1.2 mm (below) used for fat emulsification. Scale bar: 5 mm. **(C)** Cell filter (500 μm pore size) sandwiched between two Luer lock connector halves to filter the emulsified fat. Scale bar: 5 mm. **(D)** Experimental protocol of the present study. The implantation of the dorsal skinfold chamber was performed 48 h prior to nanofat injection in male (*n* = 8) and female (*n* = 8) mice. Graft vascularization was repeatedly analyzed by means of intravital fluorescence microscopy (IVM) on day 0, 3, 6, 10 and 14 after transplantation. **(E)** Overview of the dorsal skinfold chamber during nanofat injection with a 27G needle. Scale bar: 2 mm. **(F)** Higher magnification of the graft injection site within the observation window. Scale bar: 2 mm.

The inguinal subcutaneous adipose tissue (Figure 1A) of anesthetized GFP^+^ donor mice was excised, taking care not to include the inguinal lymph node. The tissue was then minced by means of a histology tissue cutter (McIlwain Tissue Chopper, CLE Co. Ltd., Gomshall, UK) to produce fat fragments of an identical volume (1 × 1 × 1 mm), which were rinsed in 0.9% NaCl solution. For emulsification, the fat was shuffled between two syringes using three female-to-female Luer lock connectors with descending internal diameters of 2.4, 1.4 and 1.2 mm and 30 passes per connector (Figure 1B). In a last step, the fat was passed through a cell filter with 500 µm pore size to remove any larger remaining fat particles or debris (Figure 1C).

### Dorsal Skinfold Chamber Model

A dorsal skinfold chamber was implanted into GFP^−^ recipient mice, as described previously in detail (Laschke et al., 2011). For this purpose, the extended skinfold was sandwiched between two titanium chamber frames (Irola Industriekomponenten GmbH & Co. KG, Schonach, Germany). One layer of skin was completely removed in a circular area of ∼15 mm in diameter. The remaining layers of striated panniculus carnosus muscle, subcutaneous tissue and skin served as the host tissue for the nanofat grafts and were sealed with a removable cover glass, which was fixed by a snap ring. Following this implantation procedure, the animals were allowed to recover for 48 h to exclude alterations of the microcirculation due to anesthesia or surgical trauma (Figure 1D). Subsequently, the recipient mice were anesthetized again, the cover glass of chamber window was removed and 10 µL of freshly prepared nanofat were injected into the subcutis underneath the panniculus carnosus muscle using a 27 G needle (Figures 1E,F). Thereafter, the chamber was closed with a new cover glass and the snap ring. The remainder of the prepared nanofat was embedded in 1% agarose for histological and immunohistochemical analyses. All animals tolerated the chamber implantation and nanofat grafts well and showed normal feeding and sleeping habits during the observation period.

### Intravital Fluorescence Microscopy

Repeated intravital fluorescence microcopy was performed directly after nanofat grafting (day 0) as well as on days 3, 6, 10 and 14 (Figure 1D). The microscopy was conducted before and after the retrobulbar injection of 0.1 ml of the blood plasma marker 5% fluorescein isothiocyanate (FITC)-labeled dextran (150,000 Da; Sigma-Aldrich, Taufkirchen, Germany) for contrast enhancement. For this purpose, the anesthetized animals were fixed on a plexiglass stage and placed under a Zeiss Axiotech fluorescence epi-illumination microscope (Zeiss, Oberkochen, Germany). The nanofat grafts were recorded with a charge-coupled device video camera (FK6990; Pieper, Schwerte, Germany) and a DVD system. By means of 5x, ×10 and ×20 long-distance objectives (Zeiss) magnifications of×115, ×230 and x460 were achieved on a 14-inch video screen (Trinitron, Sony, Tokyo, Japan).

The microscopic images were analyzed by means of the offline analysis system CapImage (Version 8.5, Zeintl, Heidelberg, Germany). The analysis included the quantitative assessment of the perfused graft surface (in % of the total graft surface). Moreover, the functional microvessel density (FMD), i.e. the overall length of all red blood cell (RBC)-perfused microvessels per observation area (in cm/cm^2^), was measured in 5 regions of interest (ROI; ×460 magnification) within the grafts. Furthermore, the fraction of ROI with GFP^+^ blood-perfused microvessels out of all ROI (in %) was assessed. In addition, microhemodynamic parameters were measured in up to 5 randomly chosen microvessels per ROI, as soon as blood perfusion could be detected. Vessel diameters (D, in µm) were measured perpendicular to the vessel path. The centerline RBC velocity (V, in µm/s) was assessed using the line shift method (De Vriese et al., 2000). The volumetric blood flow (VQ, in pL/s) was calculated from V and D as VQ = π×(D/2)^2×V/K where K = 1.6 represents the Baker-Wayland factor considering the parabolic velocity profile of blood in microvessels (Baker and Wayland, 1974). Additionally, the measured microhemodynamic parameters were used to calculate the wall shear rate (y, in s^−1^) by means of the Newtonian definition y = 8×V/D.

After the last microscopy, the animals were euthanized by cervical dislocation and the dorsal skinfold chamber preparations were carefully excised for further histological and immunohistochemical analyses.

### Histology and Immunohistochemistry

Samples of freshly generated nanofat as well as dorsal skinfold chamber preparations with nanofat grafts were fixed in paraformaldehyde, embedded in paraffin and cut into 3-µm-thick sections. Hematoxylin and eosin (HE) as well as Sirius red stainings of individual sections were performed according to standard procedures.

For the immunohistochemical detection of adipocytes, tissue sections were stained with a monoclonal rabbit anti-mouse antibody against perilipin (1:200; Cell Signaling Technology, Danvers, United States) as primary antibody followed by a goat anti-rabbit peroxidase-labeled antibody (1:100; Jackson ImmunoResearch Laboratories, West Grove, United States). The used chromogen was 3-amino-9-ethylcarbazole (Abcam, Cambridge, United Kingdom). All sections were counterstained with Mayer’s hemalum (Merck, Darmstadt, Germany).

For the immunohistochemical detection of endothelial cells, tissue sections were stained with a monoclonal rat anti-mouse antibody against CD31 (1:100; Dianova, Hamburg, Germany) as primary antibody and a goat anti-rat Alexa 555 antibody (1:100; Invitrogen, Waltham, United States) as secondary antibody. To determine the origin of the endothelial cells, the sections were additionally stained with a polyclonal GFP goat antibody (1:100; Rockland Immunochemicals Inc., Limerick, United States) followed by a donkey-anti-goat biotin-labeled antibody (1:100; Life Technologies, Carlsbad, United States) and Alexa 488-labeled streptavidin (1:50; Invitrogen). On each section, cell nuclei were stained with Hoechst 33,342 (2 μL/ml; Sigma-Aldrich) to merge images exactly.

All sections were assessed using a BX60 microscope (Olympus, Hamburg, Germany) and the imaging software cellSens Dimension 1.11 (Olympus). The number of CD31^+^ vessel segments was quantified in 3 high-power fields (HPF) per tissue specimen of freshly generated nanofat using the image processing software Fiji (open source software). In addition, we measured the fraction of CD31^+^/GFP^+^ microvessels out of all CD31^+^ microvessels in 3 HPF per nanofat graft in the dorsal skinfold chamber.

### Statistical Analysis

After testing the data for normal distribution and equal variance, differences between the two groups were analyzed by the unpaired Student’s t-test (GraphPad Prism 9; GraphPad Software, San Diego, United States). In case of non-parametric data, a Mann-Whitney rank sum test was used. All values are expressed as means ± standard error of the mean (SEM). Statistical significance was accepted for a value of *p* < 0.05.

## Results

### Characterization of Freshly Generated Nanofat

**FIGURE 2:**
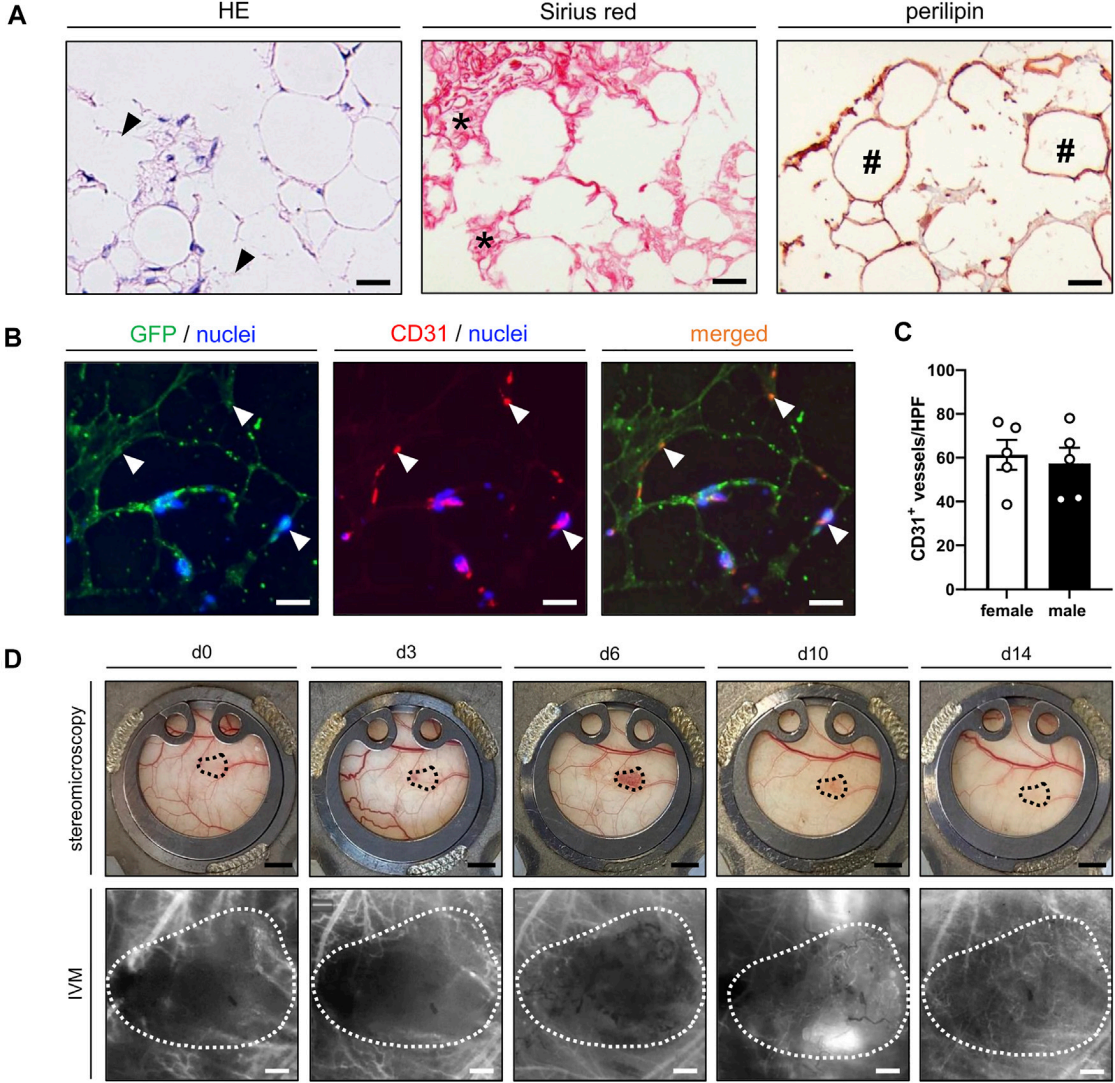
**(A)** Histological and immunohistochemical stainings of freshly isolated nanofat exhibiting remnants of burst adipocytes and cell detritus (HE; arrowheads), Sirius red^+^ collagen fibers (asterisks) and intact perilipin^+^ adipocytes (hashtags). Scale bars: 25 μm. **(B)** Immunofluorescent CD31/GFP stainings of freshly isolated nanofat containing intact vessel segments (arrowheads) in between and around adipocytes and adipocyte remnants. Cell nuclei are stained with Hoechst 33,342 (blue). Scale bars: 25 µm. **(C)** Number of CD31^+^ vessels per HPF in freshly generated nanofat from female (*n* = 5; white bars) and male (*n* = 5; black bars) donor mice. **(D)** Stereomicroscopic and intravital fluorescence microscopic (IVM) images of a nanofat graft (borders marked by dotted line) within the dorsal skinfold chamber of a female mouse on day 0, 3, 6, 10 and 14 after transplantation. Scale bars (upper panels): 2 mm; scale bars (lower panels): 200 μm. Means ± SEM.

The cellular composition and morphology of freshly generated nanofat was analyzed by means of histology and immunohistochemistry. The effects of emulsification were clearly visible by the presence of ruptured adipocytes and cellular debris throughout the grafts (Figure 2A). Of interest, the nanofat also contained significant amounts of Sirius red^+^ collagen fibers of the extracellular matrix and intact perilipin^+^ adipocytes (Figure 2A). Between these adipocytes, well preserved CD31^+^ vessel segments could be detected (Figure 2B). In freshly generated nanofat from both male and female donor mice ∼60 vessel segments per HPF could be counted (Figure 2C).

### *In Vivo* Vascularization of Grafted Nanofat

The nanofat grafts were analyzed by means of repeated stereomicroscopy and intravital fluorescence microscopy within the dorsal skinfold chambers of male and female recipient mice. All grafts survived the transplantation procedure and could be easily detected within the chamber window throughout the observation period of 14 days (Figure 2D).

**FIGURE 3:**
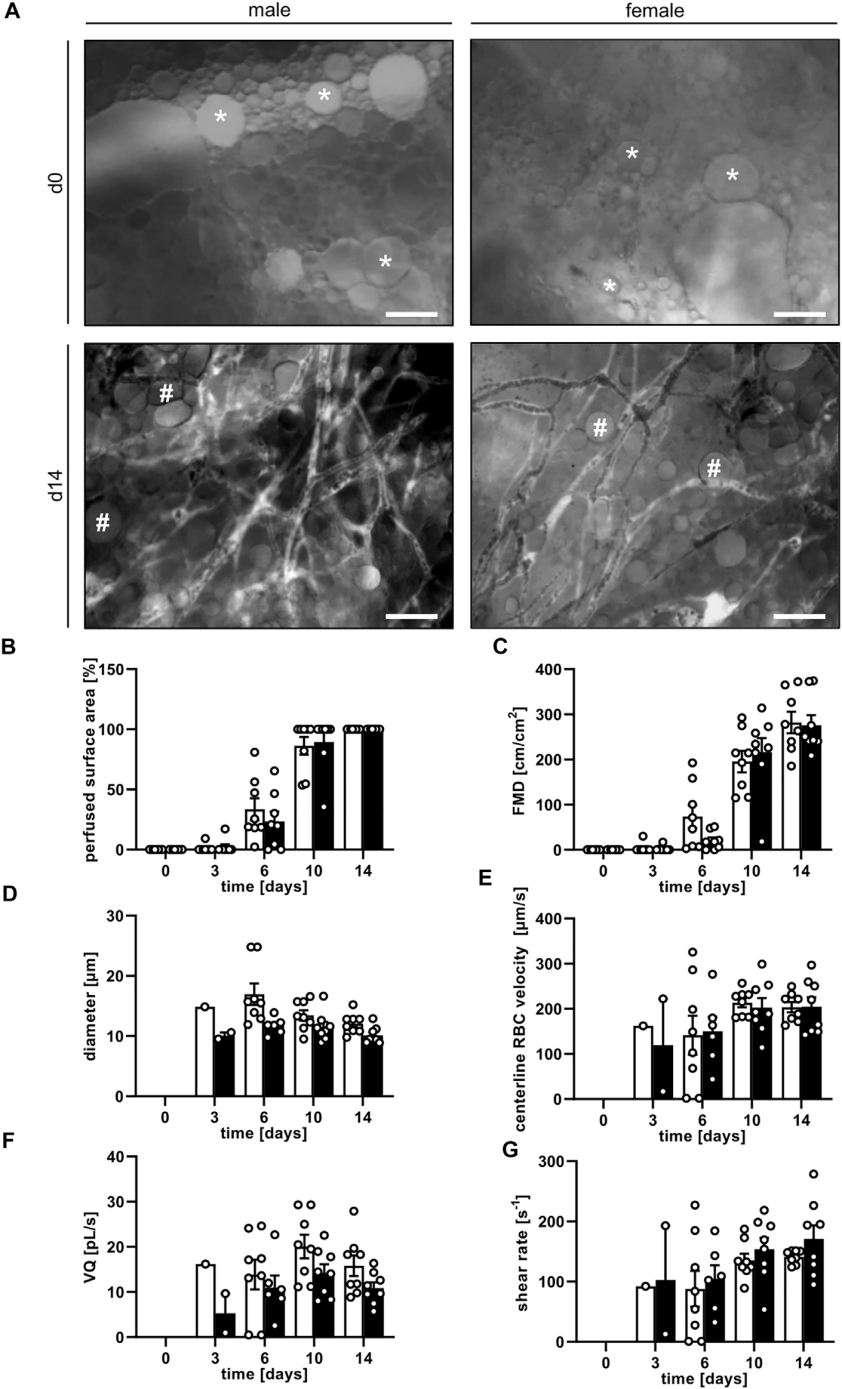
**(A)** Intravital fluorescence microscopic images of nanofat grafts in a male and a female mouse on day 0 and day 14 after transplantation. On day 0, the grafts consist of individual cells, fluid or oil vacuoles of varying sizes (asterisks) without a distinct tissue architecture. On day 14, the grafts contain newly formed, microvascular networks interspersed with adipocytes and not yet reabsorbed oil vacuoles (hashtags). Blood perfusion is evidenced by the injection of FITC-labeled dextran for contrast enhancement by staining of blood plasma. Scale bars: 50 μm. **(B–G)** Perfused surface area (**(B)**, %), FMD (**(C)**, cm/cm^2^), diameter (**(D)**, µm), centerline RBC velocity (**(E)**, µm/s), VQ (**(F)**, pL/s) and shear rate (**(G)**, s^−1^) of nanofat grafts in female (white bars; *n* = 8) and male (black bars; *n* = 8) mice on days 0, 3, 6, 10 and 14 after transplantation, as assessed by intravital fluorescence microscopy and computer-assisted image analysis. Means ± SEM.

Graft vascularization did not markedly differ between male and female mice. Directly after transplantation, the grafts of both groups exhibited a completely disintegrated tissue architecture with cells, cellular debris and oil or fluid vacuoles of varying sizes without any distinct organization (Figure 3A). First blood-perfused microvessels could already be detected on day 3. Throughout the following days, a progressively increasing number of blood-perfused microvessels was found within the grafts. These microvessels finally formed microvascular networks with an FMD of ∼280 cm/cm^2^ on day 14 (Figures 3B,C). Between these networks isolated adipocytes, small adipocyte clusters and remnants of not yet absorbed oil vacuoles could still be identified (Figure 3A). The vascularization process was slightly accelerated in female animals when compared to male mice, as indicated by a higher perfused graft area and FMD on day 6, which, however, did not prove to be significant (Figures 3B,C). The additional assessment of microhemodynamic parameters revealed that the diameter and VQ of blood-perfused microvessels remained relatively constant over time (Figures 3D,F), whereas the centerline RBC velocity and shear rate slightly increased until the end of the experiments (Figures 3E,G).

**FIGURE 4:**
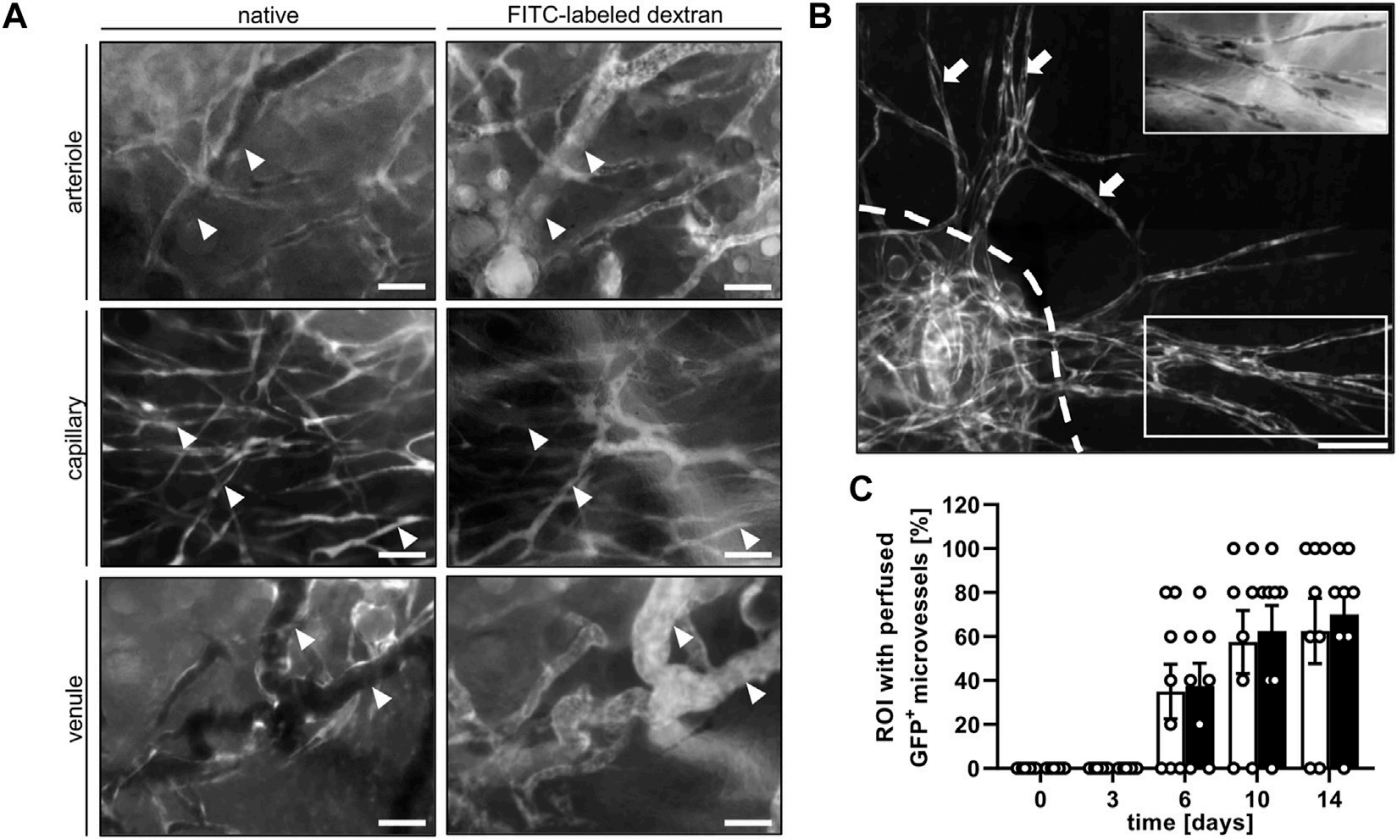
**(A)** Intravital fluorescence microscopic images of GFP^+^ arterioles (arrowheads), capillaries (arrowheads) and venules (arrowheads) before (native) and after the injection of FITC-labled dextran for contrast enhancement by staining of blood plasma on day 14 after transplantation of nanofat into a male mouse. Scale bars: 50 μm. **(B)** Intravital fluorescence microscopic images of GFP^+^ microvessel sprouts (arrows) growing out of a nanofat graft (border marked by broken line) into the surrounding host tissue on day 14 after transplantation. Insert (border marked by white line) showing the vessel sprouts after the injection of FITC-labeled dextran for contrast enhancement by staining of blood plasma. Scale bar: 50 μm. **(C)** Presence of blood-perfused GFP^+^ microvessels in the analyzed ROI of nanofat grafts in female (white bars; *n* = 8) and male (black bars; *n* = 8) mice, as assessed by intravital fluorescence microscopy. Means ± SEM.

In both groups, blood-perfused GFP^+^ microvessels could be observed in the grafted nanofat. These GFP^+^ microvessels could be further subdivided into arterioles, capillaries and venules by their distinct morphology and blood flow direction (Figure 4A). Of interest, at later observation time points the GFP^+^ microvessels grew out of the grafts into the surrounding host tissue (Figure 4B), which demonstrates their tissue vascularization capacity. The presence of GFP^+^ microvessels was detected as early as day 6 and in up to 70% of the analyzed ROI by day 14 (Figure 4C).

### Incorporation of Grafted Nanofat

**FIGURE 5:**
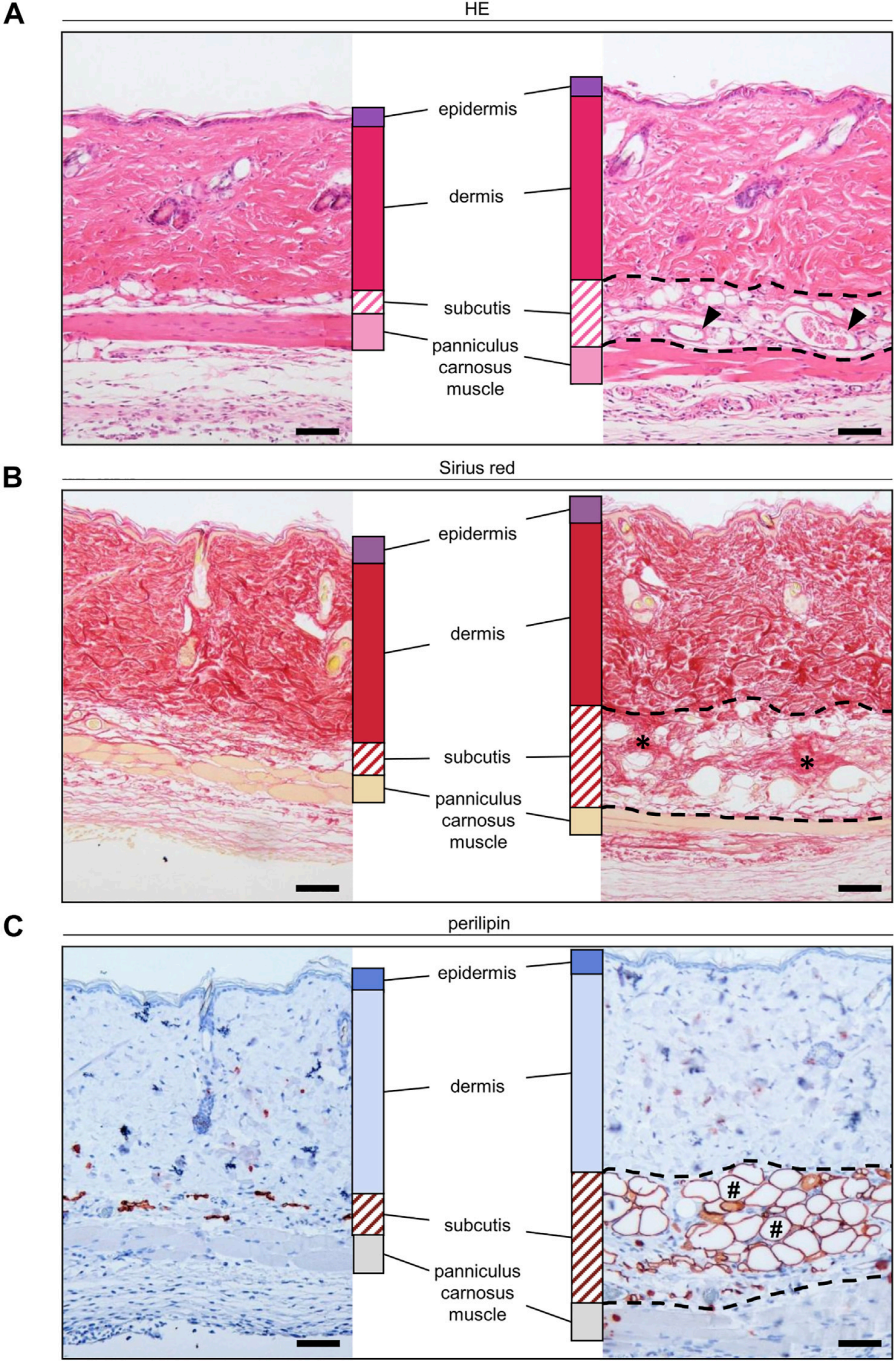
**(A–C)** Histological and immunohistochemical stainings of nanofat grafts in the subcutis (borders marked by broken line) in between the panniculus carnosus muscle and the dermis of recipient mice on day 14 after transplantation. The grafts exhibit microvessels (**(A)**, arrowheads), collagen fibers (**(B)**, asterisks) and mature adipocytes (**(C)**, hashtags). Scale bars: 50 μm.

At the end of the *in vivo* experiments, additional histological and immunohistochemical analyses were performed to examine the morphology and incorporation of the nanofat grafts. HE-stained tissue sections confirmed that the grafts were localized in the subcutis between the dermis and panniculus carnosus muscle (Figure 5A). On day 14, they still consisted of microvessels, fibrous tissue and perilipin^+^ adipocytes (Figures 5A–C).

**FIGURE 6:**
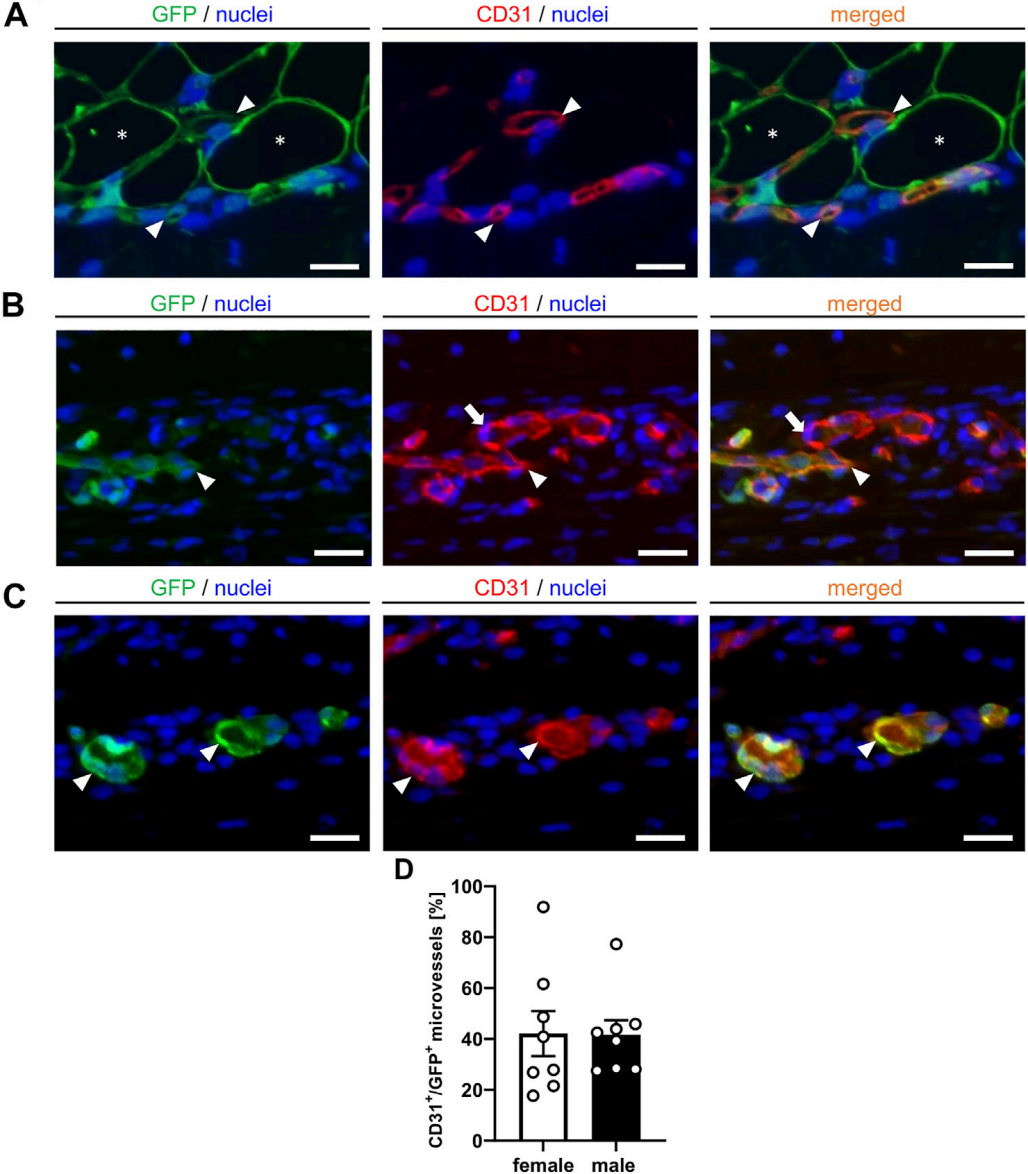
**(A–C)** Immunofluorescent CD31/GFP stainings of nanofat grafts on day 14 after transplantation into recipient mice. The grafts exhibit CD31^+^/GFP^+^ microvessels (**(A)**, arrowheads) in between GPF^+^ adipocytes (**(A)**, asterisks). These microvessels (**(B)**, arrowheads) develop interconnections to the CD31^+^/GFP^−^ microvessels (**(B)**, arrows) of the surrounding host tissue. Individual CD31^+^/GFP^+^ microvessels can also be detected outside of the nanofat grafts (**(C)**, arrowheads). Cell nuclei are stained with Hoechst 33,342 (blue). Scale bars: 10 μm. **(D)** CD31^+^/GFP^+^ microvessels (%) in nanofat grafts of female (white bars; *n* = 8) and male (black bars; *n* = 8) mice. Means ± SEM.

More detailed immunohistochemical analyses for the determination of vessel origin demonstrated that the nanofat grafts contained CD31^+^/GFP^+^ microvessels, which developed interconnections to the CD31^+^/GFP^−^ microvessels of the surrounding tissue (Figures 6A,B). In line with our intravital fluorescent microscopic findings, we further detected CD31^+^/GFP^+^ microvessels outside the grafts, resulting in an improved vascularization of the transplantation site (Figure 6C). These CD31^+^/GFP^+^ microvessels made up ∼40% of all CD31^+^ microvessels without statistical differences between male and female animals (Figure 6D).

## Discussion

Over the last decades, the indications for fat grafts in general and nanofat in particular have continuously broadened from a cosmetic to a regenerative use. In fact, the proven effectiveness of nanofat for tissue rejuvenation has led to its rapid clinical success, though its mechanisms of action remain incompletely understood (Menkes et al., 2020; Tonnard et al., 2020). So far, the effects of nanofat have been almost exclusively attributed to its high content of ASCs, which can differentiate into various cell lines (Minteer et al., 2013). In this context, Zhou et al. (2019) demonstrated a significantly upregulated fibroblast differentiation of ASCs, which was induced by co-incubation with adipose extracellular matrix. In a mouse model of skin wounds, these ASCs led to a faster wound healing when compared to extracellular matrix-free ASCs (Zhou et al., 2019). This observation demonstrates that besides ASCs, other nanofat components crucially determine its regenerative properties. In line with this view, we herein demonstrate that nanofat contains functional microvessel segments, which contribute to its capacity to boost tissue vascularization.

Several preclinical animal studies showed an increased vascularization of microfat grafts in mice with elevated serum estrogen levels (Bills et al., 2015; Mok et al., 2018; Wu et al., 2020). In line with these results, we detected a faster onset of nanofat vascularization in the dorsal skinfold chambers of female mice when compared to male animals, as indicated by a slightly larger perfused surface area and FMD of the nanofat grafts on day 6. However, this did not result in a significantly higher final FMD on day 14 after transplantation. Hence, it may be assumed that nanofat-based vascularization may also not markedly differ between female and male patients under clinical conditions.

The GFP^+^/GFP^−^ cross-over design of the present study enabled us to differentiate between GFP^+^ microvessels originating from the nanofat grafts and GFP^−^ microvessels of the host tissue. Of interest, we found that nanofat contains intact microvessel segments, which survive the transplantation procedure and develop into well-organized microvascular networks consisting of arterioles, capillaries and venules. These networks develop interconnections with the microvasculature of the host tissue, resulting in a rapid onset of blood perfusion within the grafts. In addition, they progressively grow out of the grafts and, thus, also improve the vascularization of the surrounding transplantation site. As blood-perfused, nanofat-derived GFP^+^ microvessels were detected as early as day 6 after grafting, it is not likely that they differentiated from stem cells within this time. A similar vascularization mode has already been described for adipose tissue-derived microvascular fragments (MVF) (Frueh et al., 2017; Laschke et al., 2021). These MVF can be harvested from fat samples in large amounts and have been proven to be versatile vascularization units for implanted biomaterials (Später et al., 2018; Später et al., 2020), organoids (Nalbach et al., 2021; Strobel et al., 2021) and injured tissues, such as bone and muscle (Pilia et al., 2014; Orth et al., 2018). However, their enzymatic isolation may bear the disadvantage that it requires special expensive equipment and is difficult to standardize due to dissimilar lot-to-lot enzyme activities (Trivisonno et al., 2019). Moreover, enzymatic digestion does currently not meet the requirements for minimal cell manipulation according to regulatory agencies (Raposio and Ciliberti, 2017). In contrast, the inexpensive, quick and easy mechanical generation of nanofat may overcome these hurdles. This may facilitate its widespread use in clinical practice. Although nanofat is a heterogenous mixture of cells, vessel segments and extracellular matrix compounds, it still exhibits a liquid consistency and small particle size (Cohen et al., 2017). Therefore, it may not only be used as a biological, injectable filler, but also for the seeding of biomaterial implants in order to accelerate and improve their tissue incorporation (Grünherz et al., 2019).

At the end of our *in vivo* experiments, we additionally analyzed the nanofat grafts by means of histology and immunohistochemistry. These analyses revealed that on day 14 after transplantation, the grafts still consisted of microvessels, fibrous tissue and perilipin^+^ adipocytes. Although not further examined in the present study, it would be interesting to clarify the fate of these components over longer observation periods in order to identify possible positive or negative effects associated with nanofat grafting. In this context, other long-term studies have already shown that nanofat restitutes dermal thickness after UV light-induced skin damage (Xu et al., 2018) and facilitates tissue remodeling, resulting in physiologically organized collagen and elastin fiber organization (Zheng et al., 2019).

Finally, it should be mentioned that this study also has some limitations. For intravital fluorescence microscopy, we used a conventional epi-illumination microscope with a limited depth of focus. Hence, since in our dorsal skinfold chamber model the nanofat was injected into the subcutis underneath the panniculus carnosus muscle, we may have missed microvessels in deeper layers of the grafts and the surrounding host tissue. In future studies this drawback may be overcome by means of confocal microscopy. Moreover, it may be recommended to use a plasma marker in another spectrum than the herein used FITC-labeled dextran. This would bear the advantage that the blood perfusion of GFP^+^ microvessels could be detected in another filter without an overlay of the GFP and FITC signals.

In conclusion, the present study suggests that nanofat is a potential source of functional microvessel segments with a high vascularization capacity. Even though further research is needed to confirm this finding, nanofat grafting may represent a promising approach to boost tissue vascularization. This may open the door for completely new applications of nanofat in the field of tissue engineering and regenerative medicine beyond the scope of aesthetic indications, such as the improvement of wound healing and biomaterial integration.

## Data Availability

The raw data supporting the conclusions of this article will be made available by the authors, without undue reservation.
